# Supplementary material for: Downregulation of miR-17-92 Cluster by PERK Fine-Tunes Unfolded Protein Response Mediated Apoptosis
Source: Life (Basel). 2021 Jan 6;11(1):30. doi: 10.3390/life11010030 (PMC7825066; doi:10.3390/life11010030)
Supplement: Supplementary file 1 [file life-11-00030-s001.pdf]

Downregulation of miR-17-92 cluster by PERK fine-tunes unfolded protein response mediated apoptosis

Supplementary Material

Chromatin Immunoprecipitation Assay

MCF-7 cells (3 × 10^6 cells) were transfected with wild type ATF4, NRF2 and GFP expressing plasmids. 24 hour post transfection chromatin immunoprecipitation assay was performed with a commercial kit (ChIP-IT enzymatic kit, Active Motif_Cat# 53006) according to the manufacturer's protocol. In brief, cells were treated with 1% (v/v) formaldehyde at room temperature for 10 min and then quenched with glycine at room temperature. The medium was removed, and cells were harvested in lysis buffer. Following 30 mins incubation on ice, samples were passed through a 23 gauge syringe up and down along the side of tube ~30 times to release nuclei and then centrifuged at 5000 rpm for 10 min at 4^0C to pellet nuclei. The nuclei pellet was resuspended in 1 ml digestion buffer with 5 µL PMSF and 5 µL PIC and pre-warmed for 5 min at 37^0C. For enzymatic shearing, 50 µL of 1:100 diluted shearing enzyme was added to pre-warmed nuclei, vortexed to mix and incubated at 37^0C for 30 mins. The reaction was stopped by adding 20 µL ice cold EDTA for 10 mins on ice. The sample was centrifuged at 10,000 rpm for 10 mins at 4^0C and the supernatant collected. 25 µL was kept for DNA purification and confirmation of shearing on a 1% agarose gel. The remainder was aliquoted into 250 µL volumes and stored at -80^0C (each aliquot can be used for 4 chip reactions). The sheared samples were precleared with Protein G-agarose beads at 4^0C overnight. A small amount of chromatin (10 µL) was kept as the input sample at -20^0C until the IP samples were ready. Subsequently, immunoprecipitation was conducted with anti-ATF4 antibody (Proteintech_Cat# 60035-1), anti-Nrf2 antibodies (Epitomocs_Cat# 2178-1). Normal IgG and RNA Pol II anti-bodies were used as controls (provided within the kit). Immunocomplexes were collected the following morning using Protein G-agarose beads pelleted by centrifugation and washed with low salt buffer, high salt buffer, and Tris-EDTA buffer (25 mM Tris-HCl, 150 mM NaCl, 1 mM EDTA, pH 7.2) to remove any nonspecific binding. The immunocomplexes were eluted from the beads using 50 µL of elution buffer (1 M $NaHCO_3$, 1% SDS). The cross-links of the protein-DNA complexes were reversed by adding 4 µL of 5M NaCl and 1 µL of RNAse A followed by an overnight incubation of the eluted products at 65^0C. A total of 2 µL of proteinase K (10 µg/µL) was subsequently added to the solution, and samples were incubated at 42^0C for 2 h. DNA was then purified using the spin columns provided. MiR-17-92 gene promoter sequences were amplified by PCR with miR17-92Fwd: 5'- GTGTCAATCCATTTGGGAGAG-3' and miR17-92Rev: 5'-TGGTCACAATCTTCAGTTTTAC-3'. CHOP gene promoter sequences were amplified by CHOPFwd: 5'-GGGCCAAGAAATATGGGAGT-3' and CHOPRev: 5'-TAGTCGGTCGTGAGCCTCTT-3'. Hemeoxygenase-1 gene promoter sequences were amplified by HO-1Fwd: 5'-GCTGCCCAAACCACTTCTGT-3' and HO-1Rev: 5'-GCCCTTTCACCTCCCACCTA-3'. The PCR products were analysed in a 1% agarose gel.

Supplementary Table 1. List of differentially expressed microRNAs in H9c2 cells during conditions of ER stress.

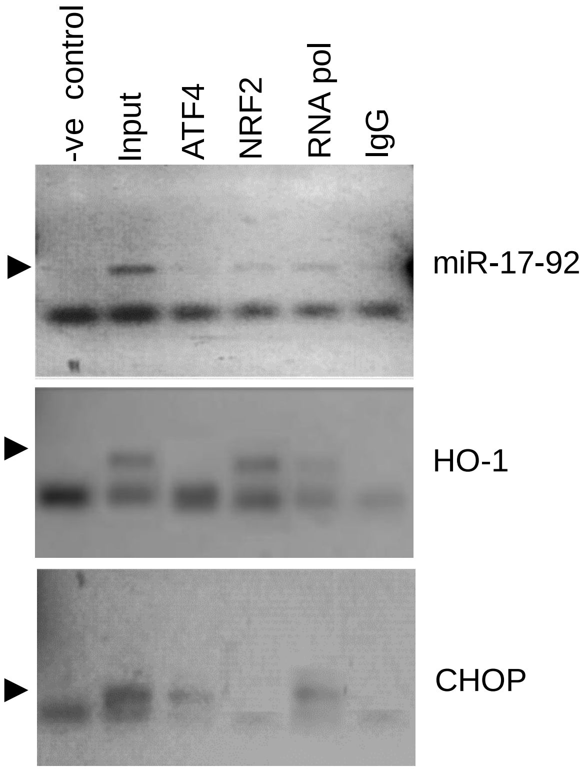

Supplementary Figure 1. MCF-7 cells were transfected with GFP, ATF4 or NRF-2 expression plasmids and ChIP assays were performed to evaluate their interaction with promoter of miR-17-92. ChIP assay was performed with antibodies directed against either ATF4 or NRF2. Mouse IgG and RNA Pol II antibodies were used as controls for the ChIP assay. DNA was extracted from the precipitates and a DNA fragment from the miR-17-92 gene promoter sequence was amplified by PCR. DNA fragments from either HO-1 or CHOP were also amplified as positive control for NRF2 and ATF4 respectively. The PCR products were resolved on agarose gels and stained with ethidium bromide. Arrows indicate the expected PCR product. -ve control is no template control for PCR. The lower band in -ve control and all the samples is likely due to formation of primer dimer.

			Group 1 control		Group 2 TG		Group 3 TM		Group 1 S01 - control-1	Group 1 S02 - control-2	Group 2 S03 - TG-1	Group 2 S04 - TG-2	Group 3 S05 - TM-1	Group 3 S06 - TM-2
No.	Reporter Name	p-value	Mean	StDev	Mean	StDev	Mean	StDev	Signal	Signal	Signal	Signal	Signal	Signal
62	rno-miR-145	7.39E-05	5,785	31	8,216	84	5,174	86	5,807	5,763	8,275	8,156	5,113	5,235
6	rno-let-7d*	2.78E-04	822	4	2,799	215	1,377	32	824	819	2,951	2,647	1,355	1,400
144	rno-miR-24	3.05E-04	8,241	176	5,475	90	5,536	89	8,116	8,365	5,538	5,411	5,473	5,599
326	rno-miR-7a	4.54E-04	334	15	448	8	822	42	345	324	454	443	852	792
75	rno-miR-16	4.78E-04	6,255	106	4,567	88	4,911	13	6,180	6,330	4,505	4,630	4,920	4,901
10	rno-let-7i	5.15E-04	10,167	10	9,843	16	9,693	33	10,160	10,173	9,854	9,832	9,717	9,670
243	rno-miR-374	6.17E-04	619	34	1,060	27	1,636	92	643	594	1,041	1,079	1,701	1,571
130	rno-miR-218	8.16E-04	488	32	388	3	859	32	511	465	386	390	882	836
154	rno-miR-27b	9.42E-04	1,239	11	1,096	16	1,314	8	1,231	1,247	1,085	1,108	1,309	1,320
152	rno-miR-27a	1.09E-03	2,607	107	1,564	3	1,865	65	2,531	2,682	1,562	1,567	1,820	1,911
167	rno-miR-29a	1.23E-03	6,335	101	4,859	60	5,165	114	6,264	6,407	4,902	4,817	5,245	5,084
60	rno-miR-143	1.28E-03	2,487	36	1,739	12	1,599	78	2,462	2,513	1,748	1,731	1,544	1,654
1	rno-let-7a	1.33E-03	16,348	343	18,755	27	20,012	178	16,591	16,105	18,736	18,774	19,886	20,138
234	rno-miR-351	1.49E-03	1,337	318	188	6	239	6	1,112	1,562	183	192	243	235
141	rno-miR-23a	1.49E-03	15,068	216	16,655	11	14,616	94	14,915	15,221	16,663	16,647	14,682	14,550
192	rno-miR-322	1.67E-03	540	119	51	2	61	13	624	456	49	52	70	52
126	rno-miR-214	1.85E-03	23,266	384	25,297	375	21,051	105	22,994	23,538	25,562	25,032	21,125	20,976
93	rno-miR-191	2.95E-03	5,388	172	4,204	81	4,200	77	5,509	5,267	4,146	4,261	4,255	4,145
349	rno-miR-99b	3.59E-03	3,242	55	3,051	29	2,241	131	3,203	3,281	3,030	3,071	2,149	2,334
82	rno-miR-181d	3.79E-03	333	30	514	7	819	89	312	354	518	509	882	756
347	rno-miR-99a	3.95E-03	1,099	69	1,651	10	1,299	21	1,148	1,050	1,643	1,658	1,284	1,314
25	rno-miR-122	4.29E-03	775	330	32	14	25	0	1,008	541	42	22	25	25
21	rno-miR-107	4.76E-03	912	10	622	7	548	49	919	906	627	617	514	583
147	rno-miR-25	4.85E-03	6,987	121	4,423	24	5,386	413	7,073	6,902	4,406	4,440	5,678	5,094
74	rno-miR-15b	5.24E-03	7,930	35	6,535	70	8,298	358	7,905	7,954	6,486	6,584	8,551	8,045
9	rno-let-7f	6.03E-03	12,072	513	14,311	247	16,145	464	12,435	11,710	14,137	14,486	16,473	15,817
29	rno-miR-125a-5p	6.04E-03	5,931	24	6,492	195	7,257	159	5,948	5,914	6,630	6,354	7,369	7,144
5	rno-let-7d	7.12E-03	12,712	501	13,986	6	15,539	6	13,067	12,358	13,982	13,990	15,543	15,534
18	rno-miR-103	7.29E-03	1,038	9	679	8	593	68	1,031	1,044	673	685	545	641
344	rno-miR-93	7.56E-03	1,339	129	754	5	702	72	1,248	1,430	758	750	650	753
104	rno-miR-199a-5p	8.46E-03	2,062	29	1,812	49	1,360	114	2,042	2,083	1,846	1,777	1,280	1,440
342	rno-miR-92a	8.93E-03	5,093	120	5,309	91	5,956	109	5,177	5,008	5,373	5,244	5,879	6,034
19	rno-miR-106b	9.01E-03	823	8	525	30	553	48	829	818	546	504	519	588
346	rno-miR-98	1.10E-02	310	6	1,185	84	1,526	564	314	306	1,125	1,244	1,924	1,127
114	rno-miR-206	1.12E-02	2,420	53	1,652	34	1,470	171	2,457	2,383	1,628	1,676	1,349	1,591
235	rno-miR-352	1.17E-02	1,224	160	1,822	51	2,156	59	1,337	1,111	1,786	1,858	2,197	2,114

181	rno-miR-30b-5p	1.25E-02	1,025	34	1,105	63	1,526	116	1,048	1,001	1,150	1,061	1,608	1,444
191	rno-miR-320	1.36E-02	2,878	203	5,026	127	3,015	400	2,735	3,022	4,936	5,116	2,732	3,298
2	rno-let-7b	1.53E-02	12,879	253	14,569	249	15,416	604	13,058	12,700	14,745	14,393	14,988	15,843
150	rno-miR-26b	1.57E-02	1,509	129	1,988	37	2,657	309	1,600	1,417	2,014	1,962	2,875	2,438
239	rno-miR-365	1.87E-02	171	51	222	28	757	214	207	135	242	202	908	605
78	rno-miR-181a	1.91E-02	2,236	121	2,721	74	2,064	104	2,150	2,321	2,668	2,773	1,990	2,138
135	rno-miR-22	2.96E-02	1,076	61	1,131	19	911	40	1,119	1,032	1,117	1,145	883	939
4	rno-let-7c	3.06E-02	15,312	359	16,876	239	17,592	668	15,566	15,058	17,045	16,707	17,119	18,065
76	rno-miR-17	3.12E-02	2,109	146	1,509	30	1,486	163	2,006	2,212	1,487	1,530	1,371	1,601
138	rno-miR-222	3.17E-02	2,761	182	1,984	5	1,756	250	2,632	2,889	1,981	1,988	1,580	1,932
23	rno-miR-10a-5p	3.67E-02	760	34	924	46	1,219	184	784	737	957	892	1,349	1,089
39	rno-miR-130a	4.01E-02	609	13	737	27	581	47	619	600	755	718	548	615
71	rno-miR-152	4.09E-02	2,333	127	2,553	63	2,167	16	2,243	2,422	2,597	2,509	2,156	2,179
7	rno-let-7e	4.11E-02	4,480	309	5,846	197	7,046	1,033	4,698	4,261	5,707	5,986	7,776	6,315
86	rno-miR-185	4.22E-02	628	20	537	9	485	44	614	643	531	544	454	516
182	rno-miR-30c	4.49E-02	2,164	37	1,923	104	2,341	116	2,190	2,138	1,996	1,849	2,423	2,259
343	rno-miR-92b	5.00E-02	2,067	32	2,215	141	2,439	21	2,089	2,044	2,315	2,116	2,454	2,424
69	rno-miR-151	5.47E-02	2,330	94	1,840	47	1,851	185	2,264	2,397	1,806	1,873	1,720	1,982
149	rno-miR-26a	5.64E-02	14,589	407	12,930	78	14,079	601	14,301	14,877	12,875	12,985	13,653	14,504
24	rno-miR-10b	5.77E-02	1,047	96	1,028	49	1,525	232	1,115	979	1,062	993	1,688	1,361
117	rno-miR-20a	6.21E-02	2,207	129	1,699	51	1,745	184	2,116	2,298	1,663	1,735	1,615	1,875
36	rno-miR-128	6.65E-02	897	25	1,001	3	1,115	102	915	879	1,004	999	1,043	1,187
137	rno-miR-221	6.96E-02	1,772	4	2,008	55	1,708	123	1,775	1,770	2,047	1,970	1,621	1,795
80	rno-miR-181b	7.75E-02	1,387	194	1,136	24	1,618	139	1,250	1,524	1,119	1,153	1,519	1,716
121	rno-miR-21	7.99E-02	30,343	716	29,775	420	31,566	234	29,836	30,849	29,478	30,072	31,731	31,401
70	rno-miR-151*	8.27E-02	728	30	802	7	674	49	706	749	796	807	639	709

Following transcripts are statistically significant but have low signals (signal < 500)

90	rno-miR-18a	1.14E-03	272	14	116	5	149	10	262	281	119	112	143	156
127	rno-miR-215	3.30E-03	24	2	47	1	41	2	22	25	47	46	43	39
163	rno-miR-296*	5.56E-03	103	7	182	12	96	8	98	108	190	173	90	101
41	rno-miR-132	6.98E-03	271	12	367	4	302	11	262	280	370	364	310	294
140	rno-miR-224	8.17E-03	25	5	90	13	46	0	22	29	99	80	46	46
233	rno-miR-350	9.78E-03	372	16	297	12	375	2	383	361	305	288	377	374
56	rno-miR-140*	1.06E-02	275	16	280	21	186	9	264	286	265	295	179	192
156	rno-miR-28*	1.27E-02	214	17	346	17	288	19	227	202	359	334	302	274
55	rno-miR-140	1.30E-02	37	4	20	2	27	1	39	34	18	21	28	27
298	rno-miR-532-3p	1.31E-02	88	2	213	4	148	31	89	86	215	210	170	126
54	rno-miR-139-5p	1.31E-02	42	2	30	3	44	1	43	41	29	32	45	43
157	rno-miR-290	1.42E-02	68	30	439	10	191	30	47	89	432	446	171	212
334	rno-miR-877	1.48E-02	138	1	94	0	110	11	139	138	94	94	103	118

33	rno-miR-126	1.55E-02	70	6	181	13	164	40	74	66	190	172	192	136
333	rno-miR-874	1.55E-02	36	1	143	28	76	21	35	37	123	163	60	91
303	rno-miR-542-3p	2.03E-02	72	14	35	2	50	3	81	62	36	33	52	48
3	rno-let-7b*	2.10E-02	70	7	140	22	100	5	65	75	155	124	103	97
43	rno-miR-133b	2.35E-02	130	5	73	2	83	14	126	133	72	74	93	73
131	rno-miR-218*	2.38E-02	44	5	29	1	28	2	48	40	28	29	26	29
315	rno-miR-674-3p	2.42E-02	247	21	190	5	181	9	233	262	193	187	174	187
178	rno-miR-30a	2.57E-02	366	21	344	8	457	31	381	351	349	338	479	435
220	rno-miR-342-5p	2.74E-02	98	5	74	5	79	2	94	101	78	70	78	80
153	rno-miR-27a*	2.85E-02	19	2	37	6	23	2	18	21	32	41	21	24
148	rno-miR-25*	3.09E-02	127	16	90	3	87	0	115	138	88	93	87	88
142	rno-miR-23a*	3.81E-02	277	38	386	16	230	28	250	304	397	375	211	250
207	rno-miR-331	3.95E-02	97	15	133	8	83	4	87	108	127	138	81	86
184	rno-miR-30c-2*	3.98E-02	40	2	47	4	36	0	39	42	50	45	37	36
88	rno-miR-187	4.29E-02	50	2	35	3	37	4	49	51	37	33	41	34
136	rno-miR-22*	4.36E-02	57	2	97	4	92	20	58	55	100	95	106	78
125	rno-miR-212	4.36E-02	28	2	42	7	39	0	29	26	47	37	39	38
91	rno-miR-190	4.54E-02	33	7	14	0	26	7	38	29	14	14	31	21
42	rno-miR-133a	4.69E-02	111	8	66	5	77	14	105	117	69	63	86	67
208	rno-miR-333	4.78E-02	91	8	134	10	147	23	96	85	141	127	164	131
284	rno-miR-484	5.41E-02	73	1	70	5	58	3	72	73	66	74	56	61
317	rno-miR-708	5.80E-02	24	4	49	13	42	3	21	26	58	40	45	40
106	rno-miR-19b	6.04E-02	116	15	200	14	192	41	106	127	210	190	221	163
297	rno-miR-505	6.51E-02	190	16	250	6	248	27	201	178	254	246	267	230
89	rno-miR-188	6.59E-02	45	8	100	16	70	18	39	50	89	112	57	83
118	rno-miR-20a*	6.64E-02	38	3	34	4	26	2	36	41	31	37	27	24
120	rno-miR-20b-5p	6.77E-02	180	12	174	26	297	61	171	188	192	156	340	253
176	rno-miR-301a	6.94E-02	27	5	51	6	47	10	24	30	55	46	54	39
292	rno-miR-497	8.00E-02	36	4	24	0	35	7	39	34	23	24	30	39
40	rno-miR-130b	8.03E-02	43	4	26	6	30	1	40	45	22	30	31	29
280	rno-miR-466b	8.22E-02	25	11	66	9	45	7	33	18	60	73	50	40
331	rno-miR-872*	8.97E-02	53	15	87	0	105	23	64	43	87	87	121	89
203	rno-miR-329	9.15E-02	35	7	33	2	21	4	30	41	31	34	19	24
179	rno-miR-30a*	9.55E-02	42	0	65	17	70	4	42	42	77	52	73	67
15	rno-miR-101a	9.59E-02	38	7	26	4	37	1	43	33	28	23	37	38
63	rno-miR-146a	9.81E-02	30	4	49	7	39	6	28	33	54	44	44	35

Following transcripts are statistically insignificant (p-value > .1)

172	rno-miR-29c	1.01E-01	139	6	322	51	603	389	143	135	286	358	878	327
193	rno-miR-322*	1.03E-01	545	36	672	8	575	55	570	520	667	678	536	614
133	rno-miR-219-2-3p	1.03E-01	40	6	30	2	29	1	45	36	29	32	30	28

188	rno-miR-30e*	1.12E-01	29	4	35	1	39	4	26	32	36	35	42	37
161	rno-miR-292-5p	1.16E-01	42	14	81	10	47	8	32	52	88	74	42	53
189	rno-miR-31	1.16E-01	1,384	142	1,475	36	1,067	175	1,284	1,484	1,501	1,450	944	1,191
269	rno-miR-425	1.42E-01	250	22	158	39	164	29	234	265	131	186	143	184
229	rno-miR-34a	1.46E-01	514	109	360	14	354	46	436	591	350	371	321	386
119	rno-miR-20b-3p	1.55E-01	39	5	26	5	28	4	42	35	29	22	26	31
72	rno-miR-153	1.56E-01	26	9	16	3	30	4	20	33	14	18	27	33
313	rno-miR-672	1.57E-01	33	6	43	2	43	4	29	37	44	41	46	40
252	rno-miR-378*	1.60E-01	31	2	41	6	45	8	32	30	46	37	39	51
316	rno-miR-674-5p	1.63E-01	375	52	375	9	304	20	338	412	381	368	290	318
232	rno-miR-34c*	1.71E-01	790	26	964	52	706	144	771	809	927	1,001	604	808
266	rno-miR-412	1.74E-01	17	4	22	2	28	6	15	20	21	24	24	33
202	rno-miR-328	1.78E-01	174	23	204	22	158	9	158	190	220	189	152	164
32	rno-miR-125b-5p	1.85E-01	20,264	172	19,745	156	20,113	291	20,142	20,386	19,855	19,635	20,319	19,908
11	rno-let-7i*	1.87E-01	34	1	29	6	25	0	34	33	33	25	25	25
103	rno-miR-199a-3p	2.03E-01	17,664	335	18,126	5	18,048	123	17,428	17,901	18,122	18,130	17,961	18,136
251	rno-miR-378	2.05E-01	240	30	196	21	180	25	219	262	181	211	162	198
155	rno-miR-28	2.08E-01	440	10	414	16	425	1	447	433	402	425	425	426
276	rno-miR-450a	2.08E-01	537	75	551	8	783	203	590	484	557	545	926	639
320	rno-miR-743a	2.09E-01	18	3	26	7	30	6	20	16	31	21	26	34
28	rno-miR-125a-3p	2.11E-01	86	1	102	11	92	5	87	86	109	94	88	96
212	rno-miR-338	2.15E-01	30	4	19	6	19	3	26	33	14	23	21	18
183	rno-miR-30c-1*	2.17E-01	23	8	46	11	37	11	17	29	54	38	28	45
323	rno-miR-760-3p	2.27E-01	60	8	62	3	51	5	66	55	64	60	54	47
296	rno-miR-503	2.32E-01	50	22	22	9	26	5	65	34	16	29	22	29
194	rno-miR-323	2.33E-01	22	6	34	4	24	5	18	27	31	36	21	27
214	rno-miR-339-3p	2.37E-01	36	0	31	12	22	1	36	36	39	22	22	23
159	rno-miR-291a-5p	2.48E-01	27	6	31	9	19	2	22	31	37	25	20	18
187	rno-miR-30e	2.50E-01	58	10	77	7	83	20	51	65	72	81	98	69
77	rno-miR-17-3p	2.50E-01	44	4	37	4	44	4	47	42	34	40	47	42
310	rno-miR-652	2.50E-01	59	6	78	15	76	8	64	55	68	89	70	82
96	rno-miR-193*	2.55E-01	33	2	41	10	49	9	32	34	33	48	55	42
12	rno-miR-1	2.61E-01	102	34	95	2	171	63	126	77	96	94	216	126
213	rno-miR-338*	2.62E-01	36	5	28	6	23	7	32	39	24	32	18	28
14	rno-miR-100	2.68E-01	837	82	896	24	957	51	895	779	880	913	993	921
205	rno-miR-330	2.69E-01	25	11	14	1	19	3	18	33	13	14	17	21
160	rno-miR-292-3p	3.02E-01	29	0	34	10	22	5	29	30	41	27	26	19
146	rno-miR-24-2*	3.05E-01	230	44	284	5	255	13	261	199	287	280	246	264
97	rno-miR-194	3.07E-01	72	3	70	5	85	14	74	70	73	66	95	75
53	rno-miR-139-3p	3.14E-01	38	3	26	9	35	2	40	36	20	33	36	33

305	rno-miR-543	3.15E-01	18	2	26	1	28	10	17	20	25	27	20	35
22	rno-miR-10a-3p	3.18E-01	27	12	41	13	48	10	35	18	50	32	55	41
143	rno-miR-23b	3.22E-01	13,077	360	13,368	138	12,914	192	12,822	13,332	13,465	13,270	12,778	13,050
185	rno-miR-30d	3.24E-01	846	62	974	40	878	97	802	890	1,002	945	810	947
294	rno-miR-500	3.24E-01	32	16	54	8	43	1	44	20	48	59	43	42
31	rno-miR-125b-3p	3.30E-01	33	6	35	6	42	1	37	29	39	30	43	42
95	rno-miR-193	3.31E-01	69	7	59	13	45	17	64	75	50	69	57	33
85	rno-miR-184	3.40E-01	35	2	24	9	29	2	36	34	30	17	31	28
324	rno-miR-760-5p	3.43E-01	29	3	33	4	26	5	27	31	36	30	23	29
52	rno-miR-138*	3.49E-01	39	0	39	5	44	3	39	39	36	42	46	42
165	rno-miR-298	3.49E-01	58	11	70	5	57	6	66	51	73	66	53	61
67	rno-miR-148b-5p	3.54E-01	39	2	36	1	37	2	41	37	35	37	38	35
332	rno-miR-873	3.57E-01	36	2	26	7	23	10	38	35	31	22	30	16
215	rno-miR-339-5p	3.57E-01	37	2	36	3	33	3	36	38	34	38	31	35
105	rno-miR-19a	3.66E-01	28	3	32	2	32	3	25	30	34	31	34	29
98	rno-miR-195	3.69E-01	318	31	286	21	389	105	340	297	271	301	463	314
166	rno-miR-299	3.76E-01	45	8	60	8	54	12	39	50	54	65	45	62
247	rno-miR-376b-3p	3.78E-01	30	3	19	9	24	4	28	32	13	26	27	21
49	rno-miR-136*	3.83E-01	30	2	22	8	26	3	29	32	27	17	29	24
79	rno-miR-181a*	3.92E-01	50	16	37	2	36	5	38	61	35	38	39	32
58	rno-miR-142-3p	3.99E-01	33	0	23	11	29	1	33	34	30	15	30	28
222	rno-miR-344-3p	4.02E-01	30	7	23	1	22	6	25	35	23	24	17	26
198	rno-miR-325-3p	4.03E-01	37	8	39	12	50	4	31	42	31	47	53	47
329	rno-miR-871	4.03E-01	29	8	20	5	27	5	35	23	16	24	31	24
134	rno-miR-219-5p	4.07E-01	35	6	37	10	49	11	31	40	30	44	41	57
132	rno-miR-219-1-3p	4.13E-01	36	8	25	11	35	4	30	42	32	17	32	38
44	rno-miR-134	4.21E-01	43	2	39	3	29	15	45	41	41	37	40	19
321	rno-miR-743b	4.31E-01	18	3	27	15	30	4	20	15	38	16	27	33
219	rno-miR-342-3p	4.33E-01	195	17	207	21	260	76	183	207	222	193	313	206
108	rno-miR-200b	4.36E-01	35	7	30	3	38	5	29	40	32	27	34	41
51	rno-miR-138	4.43E-01	42	14	29	1	31	9	52	32	30	28	37	24
196	rno-miR-324-3p	4.49E-01	161	55	161	8	120	22	123	200	166	155	105	136
236	rno-miR-361	4.52E-01	1,635	119	1,812	6	1,591	244	1,551	1,720	1,808	1,817	1,418	1,763
291	rno-miR-495	4.55E-01	30	4	18	14	26	0	33	27	8	28	26	26
312	rno-miR-671	4.55E-01	37	11	41	18	24	9	29	45	29	54	18	31
231	rno-miR-34c	4.58E-01	80	32	54	15	53	10	58	103	44	65	46	60
206	rno-miR-330*	4.68E-01	49	13	38	7	41	3	40	58	33	43	39	43
286	rno-miR-487b	4.72E-01	23	6	22	0	32	11	28	19	22	22	24	40
199	rno-miR-325-5p	4.84E-01	37	9	24	7	26	12	31	43	20	29	35	17
122	rno-miR-21*	4.85E-01	27	5	27	5	32	3	23	30	31	23	35	30

158	rno-miR-291a-3p	4.88E-01	30	4	31	2	27	0	27	33	32	29	27	27
64	rno-miR-146b	4.89E-01	135	22	152	14	155	11	150	119	162	142	162	147
287	rno-miR-488	4.91E-01	19	6	17	14	31	8	23	15	7	26	25	36
73	rno-miR-154	4.95E-01	35	5	23	12	27	6	39	31	14	32	31	23
244	rno-miR-375	4.95E-01	16	4	17	4	100	119	13	19	15	20	16	184
47	rno-miR-135b	5.01E-01	32	3	23	8	20	12	34	29	29	18	28	11
345	rno-miR-96	5.01E-01	16	4	25	11	17	0	14	19	33	17	17	17
180	rno-miR-30b-3p	5.08E-01	28	1	38	13	32	4	27	28	47	29	29	35
109	rno-miR-200c	5.11E-01	44	1	40	5	56	21	45	43	36	43	40	71
113	rno-miR-205	5.12E-01	29	8	21	9	28	2	23	35	15	27	29	27
299	rno-miR-532-5p	5.17E-01	51	8	60	3	61	11	57	45	58	62	53	69
290	rno-miR-494	5.17E-01	27	6	41	16	35	7	32	23	29	53	30	39
8	rno-let-7e*	5.21E-01	29	10	32	1	36	2	35	22	33	31	38	34
304	rno-miR-542-5p	5.24E-01	33	8	31	0	43	15	38	27	31	31	33	53
38	rno-miR-129*	5.32E-01	42	12	33	4	41	6	50	34	36	31	37	45
57	rno-miR-141	5.32E-01	36	10	24	13	28	0	43	29	15	33	28	28
201	rno-miR-327	5.34E-01	40	16	53	11	39	8	29	52	46	61	33	45
45	rno-miR-135a	5.35E-01	36	2	26	12	22	14	37	34	35	18	32	12
87	rno-miR-186	5.38E-01	86	5	70	12	84	22	83	90	79	62	99	68
281	rno-miR-466c	5.39E-01	19	15	21	3	31	5	30	8	24	19	34	27
81	rno-miR-181c	5.40E-01	42	7	47	1	48	5	38	47	46	47	52	45
162	rno-miR-296	5.46E-01	59	2	63	12	53	9	61	57	71	54	59	47
211	rno-miR-337	5.52E-01	26	12	18	3	24	4	17	34	16	20	27	21
164	rno-miR-297	5.54E-01	17	6	23	13	27	0	21	13	32	14	27	27
115	rno-miR-207	5.62E-01	57	8	49	10	48	5	51	63	41	56	44	51
139	rno-miR-223	5.76E-01	54	12	63	10	51	8	45	62	55	70	46	57
288	rno-miR-489	5.79E-01	33	3	24	13	29	1	35	30	14	33	29	28
197	rno-miR-324-5p	5.83E-01	139	45	113	4	107	19	107	171	116	110	94	121
123	rno-miR-210	5.89E-01	28	5	34	3	30	8	24	31	37	32	36	24
295	rno-miR-501	5.91E-01	30	12	37	1	29	4	38	22	36	37	32	26
285	rno-miR-485	5.95E-01	25	6	22	6	29	7	29	21	26	18	24	35
13	rno-miR-1*	6.13E-01	46	3	39	12	42	0	48	44	47	31	43	42
112	rno-miR-204*	6.18E-01	32	4	31	5	25	11	35	29	27	34	33	18
99	rno-miR-196a	6.37E-01	27	4	26	10	33	7	24	30	33	19	38	28
101	rno-miR-196b	6.58E-01	42	1	41	8	46	6	43	41	46	35	50	42
59	rno-miR-142-5p	6.65E-01	31	5	23	1	24	14	27	34	23	24	34	14
128	rno-miR-216a	6.75E-01	29	2	34	9	35	7	30	27	40	27	30	40
17	rno-miR-101b	6.85E-01	50	14	54	7	61	13	60	40	49	59	70	52
84	rno-miR-183	6.91E-01	30	6	24	9	29	5	25	34	30	18	33	25
107	rno-miR-200a	7.00E-01	33	13	29	3	26	3	24	42	31	26	28	23

111	rno-miR-204	7.03E-01	28	9	31	7	24	2	22	34	25	36	26	23
100	rno-miR-196a*	7.06E-01	35	4	31	7	33	3	38	33	26	36	35	30
268	rno-miR-423	7.28E-01	28	10	38	12	29	15	21	35	29	47	18	40
311	rno-miR-664	7.29E-01	37	2	44	12	39	3	36	39	35	52	37	41
129	rno-miR-217	7.29E-01	30	3	32	6	34	5	28	32	37	27	31	38
259	rno-miR-383	7.45E-01	25	13	30	4	23	7	16	35	28	33	18	29
221	rno-miR-343	7.64E-01	37	21	45	15	33	3	22	52	34	55	31	35
16	rno-miR-101a*	7.65E-01	31	2	28	2	28	9	32	30	29	26	34	22
283	rno-miR-483	7.65E-01	60	16	67	7	60	10	71	48	62	73	53	67
83	rno-miR-182	7.72E-01	41	14	43	5	37	1	31	51	40	46	37	37
61	rno-miR-144	7.79E-01	26	12	17	1	22	15	35	17	18	16	33	11
328	rno-miR-7b	7.80E-01	25	0	25	12	29	4	25	25	17	33	32	26
102	rno-miR-196c	8.08E-01	28	0	28	1	25	11	28	28	27	29	33	17
110	rno-miR-203	8.10E-01	31	9	31	2	33	0	24	37	30	33	34	33
50	rno-miR-137	8.11E-01	24	3	19	7	25	15	26	22	24	14	36	15
210	rno-miR-336	8.11E-01	63	18	51	37	58	18	50	75	25	77	45	71
68	rno-miR-150	8.22E-01	59	1	60	6	61	3	58	59	55	64	64	59
224	rno-miR-345-3p	8.27E-01	39	11	40	6	34	13	31	47	35	44	25	44
171	rno-miR-29b-2*	8.29E-01	33	1	30	11	30	6	34	33	37	22	26	35
200	rno-miR-326	8.43E-01	28	17	29	11	22	7	16	40	36	21	27	16
35	rno-miR-127	8.46E-01	31	9	26	9	28	3	37	25	33	20	26	31
273	rno-miR-434	8.57E-01	27	17	27	10	21	10	14	39	20	35	14	27
218	rno-miR-341	8.57E-01	47	21	53	1	46	19	32	62	52	54	32	59
20	rno-miR-106b*	8.59E-01	114	36	100	11	104	5	139	88	108	91	108	101
26	rno-miR-124	8.63E-01	32	7	36	10	34	0	37	27	43	29	34	34
94	rno-miR-192	8.63E-01	91	4	90	13	94	7	93	88	80	99	99	89
27	rno-miR-124*	8.85E-01	31	4	33	7	31	4	33	28	38	29	29	34
327	rno-miR-7a*	8.92E-01	45	2	51	2	51	22	46	43	49	52	66	35
302	rno-miR-541	8.96E-01	25	4	22	3	23	17	28	22	20	24	11	35
30	rno-miR-125b*	8.99E-01	50	1	49	7	50	1	51	49	54	43	51	49
37	rno-miR-129	9.22E-01	41	11	43	12	38	9	48	33	34	51	32	45
301	rno-miR-540	9.22E-01	27	2	31	17	26	18	28	26	18	43	13	39
145	rno-miR-24-1*	9.30E-01	30	13	28	5	33	12	21	39	25	32	24	42
66	rno-miR-148b-3p	9.31E-01	126	10	116	9	131	53	119	133	122	109	169	93
124	rno-miR-211	9.31E-01	28	2	27	8	28	3	27	29	33	22	27	30
226	rno-miR-346	9.40E-01	39	6	44	3	46	24	35	44	42	46	29	63
289	rno-miR-493	9.41E-01	23	3	26	19	26	1	26	21	12	39	27	25
308	rno-miR-598-3p	9.46E-01	23	4	20	10	24	17	26	20	13	27	11	36
330	rno-miR-872	9.52E-01	159	22	155	2	159	11	174	144	156	154	166	152
262	rno-miR-409-3p	9.71E-01	52	19	53	9	54	12	38	66	47	59	46	63

225	rno-miR-345-5p	9.71E-01	103	26	104	47	110	33	85	122	71	137	87	133
227	rno-miR-347	9.96E-01	33	19	33	11	32	10	20	47	25	41	25	39
173	rno-miR-29c*	9.98E-01	29	11	29	8	30	9	22	37	35	23	23	36
325	rno-miR-770	9.98E-01	30	13	30	10	29	1	39	21	37	23	29	29
177	rno-miR-301b	9.99E-01	21	0	23	15	21	6	21	21	34	13	25	17
